# Risk factors for second primary neoplasia of esophagus in newly diagnosed head and neck cancer patients: a case–control study

**DOI:** 10.1186/1471-230X-13-154

**Published:** 2013-10-25

**Authors:** Chen-Shuan Chung, Li-Jen Liao, Wu-Chia Lo, Yueh-Hung Chou, Yi-Chen Chang, Yu-Chin Lin, Wei-Fan Hsu, Pei-Wei Shueng, Tzong-Hsi Lee

**Affiliations:** 1 Departments of Internal Medicine, Division of Gastroenterology and Hepatology, Far Eastern Memorial Hospital, No. 21, Nan-Ya South Road, Section 2, Ban-Ciao, 22060 New Taipei City, Taiwan; 2 Otolaryngology, Far Eastern Memorial Hospital, Banciao District, New Taipei City, Taiwan; 3 Graduate Institute of Epidemiology and Preventive Medicine, College of Public Health, National Taiwan University, Taipei, Taiwan; 4 Anatomical Pathology, Far Eastern Memorial Hospital, Banciao District, New Taipei City, Taiwan; 5 Surgery, Far Eastern Memorial Hospital, Banciao District, New Taipei City, Taiwan; 6 Medical Oncology, Far Eastern Memorial Hospital, Banciao District, New Taipei City, Taiwan; 7 Radiation Oncology, Far Eastern Memorial Hospital, Banciao District, New Taipei City, Taiwan

**Keywords:** Image-enhanced endoscopy, Narrow-band imaging, Second primary tumor, Esophageal cancer, Head and neck cancer

## Abstract

**Background:**

The prevalence of esophageal neoplasia in head and neck (H&N) cancer patients is not low; however, routine esophageal surveillance is not included in staging of newly-diagnosed H&N cancers. We aimed to investigate the risk factors for synchronous esophageal neoplasia and the impact of endoscopy on management of H&N cancer patients.

**Methods:**

A total of 129 newly diagnosed H&N cancer patients who underwent endoscopy with white-light imaging, narrow-band imaging (NBI) with magnifying endoscopy (ME), and chromoendoscopy with 1.5% Lugol’s solution, before definite treatment were enrolled prospectively.

**Results:**

60 esophageal lesions were biopsied from 53 (41.1%) patients, including 11 low-grade, 14 high-grade intraepithelial neoplasia and 12 invasive carcinoma in 30 (23.3%) patients. Alcohol consumption [odds ratio (OR) 5.90, 95% confidence interval (CI) 1.23-26.44], advanced stage (stage III and IV) of index H&N cancers (OR 2.98, 95% CI 1.11-7.99), and lower body mass index (BMI) (every 1-kg/m^2^ increment with OR 0.87, 95% CI 0.76-0.99) were independent risk factors for synchronous esophageal neoplasia. NBI with ME was the ideal screening tool (sensitivity, specificity, and accuracy of 97.3%, 94.1%, and 96.3%, respectively, for detection of dysplastic and cancerous esophageal lesions). The treatment strategy was modified after endoscopy in 20 (15.5%) patients. The number needed to screen was 6.45 (95% CI 4.60-10.90).

**Conclusions:**

NBI-ME surveillance of esophagus should be done in newly-diagnosed H&N cancer patients, especially those with alcohol drinking, lower BMI, and advanced stage of primary tumor.

## Background

Squamous cell carcinomas of the head and neck (H&N) region and the esophagus are common dismal malignancies globally, especially in the Western Pacific regions where carcinogen uses such as drinking alcohol, cigarette smoking and betel quid chewing are prevalent [1]. Mucosa of the upper aerodigestive tract (UADT) may be exposed to common carcinogens and at risk of early molecular alterations without histopathological changes, followed by malignant transformation [2]. According to population-based analyses, the risk and incidence of second primary cancers of the index head and neck, or esophagus are quite high [3-5], and without early detection, synchronous or metachronous carcinogenesis may lead to poor prognosis despite multidisciplinary treatment of the primary cancers [6-8]. Even with traditional panendoscopy screening for synchronous esophageal cancer, the treatment result was still poor [6].

Recent advances in image-enhanced endoscopy (IEE) have enabled precancerous or early cancerous lesions visible more easily under endoscopic examination [9]. Using IEE examination, especially chromoendoscopy with Lugol’s solution and narrow-band imaging (NBI) system with high-resolution magnifying endoscopy (ME), dysplastic or cancerous lesions, and tumor invasion could be well delineated [9-17]. The prevalence of high grade intraepithelial neoplasia or invasive carcinoma of the esophagus in population at high risk, such as alcoholics or H&N cancer patients, is around 3.2 to 28% by IEE screening [11]. To triage and allocate H&N cancer patients at higher risk for esophageal neoplasia to surveillance program becomes important and cost-effective. However, routine application of endoscopic surveillance of esophagus is not a consensus nowadays, and which population benefit from the screening policy has not been well defined.

Although previous studies have found certain risk factors for synchronous esophageal neoplasia in H&N cancer patients [12,13], there was no prospective study using routine application of NBI-ME and Lugol’s chromoendoscopy screening with standardized pathological classification. The aim of this prospective study was to determine the prevalence and risk factors for synchronous esophageal neoplasia and the impact of routine IEE screening on the decision making for the management of newly diagnosed H&N cancer patients.

## Methods

### Study population and data collection

We prospectively recruited 168 adults older than 20-year-old who had newly diagnosed H&N cancers that were confirmed by two otolaryngologists (L.-J. L., W.-C. L.) from March 2010 to March 2012 at the Far Eastern Memorial Hospital in New Taipei City, Taiwan. We excluded patients with salivary gland tumors, who needed emergent surgery for compromised airways or tumor bleeding, allergic history to iodine and pregnant. A total of 129 patients were referred to gastroenterologists for IEE screening before treatment. The study population was separated into two groups: H&N cancer patients with, and without synchronous esophageal neoplasia. Demographic characteristics, including age, gender, body mass index (BMI), and the status of habitual use of common carcinogens for UADT cancers, including drinking alcohol, cigarette smoking, and betel quid chewing, were gathered. The cumulative lifetime exposure was calculated by multiplying the frequency and duration, and further categorized into these three levels (Table 1 footnotes). All the participants provided written informed consent before endoscopic examination. This study was approved by the Research Ethics Review Committee of Far Eastern Memorial Hospital (FEMH IRB-101022-E).

**Table 1:** Demographic characteristics and risk assessment of the study population

	Presence of esophageal neoplasia^*^(n=30) No. of patients (%)	Absence of esophageal neoplasia^*^(n=99) No. of patients (%)	**OR (95% CI)**	***P*-value**
**Age** (years, mean±SD)	58.76 ± 9.20	55.63 ± 9.99	1.03 (0.99-1.08)	0.130
<45	1 (3.3)	12 (12.1)	referent	
45~54	10 (33.3)	40 (40.4)	3.00 (0.34-26.82)	
≧55	19 (63.3)	47 (47.5)	4.85 (0.56-41.94)	0.077
**Sex**				
Female	2 (6.7)	5 (5.1)	referent	
Male	28 (93.3)	94 (94.9)	0.74 (0.14-4.05)	0.733
**BMI** (kg/m^2^, mean±SD)	21.64 ± 3.37	24.30 ± 4.85	0.86 (0.77-0.96)^+^	0.008
**Location of H&N cancer**				
Oral cavity	8 (26.7)	55 (55.6)	Referent	
Oropharynx	6 (20)	16 (16.2)	1.50 (0.34-6.59)	0.591
Hypopharynx	12 (40)	20 (20.2)	4.52 (1.46-13.99)	0.009
Larynx	4 (13.3)	4 (4.0)	5.70 (1.08-29.99)	0.040
Nasopharynx	0	4 (4.0)	NA	NA
**Stage of H&N cancer**				
I	4 (13.3)	19 (19.2)	Referent	
II	4 (13.3)	14 (14.1)		
III	4 (13.3)	12 (12.1)	2.30 (0.98-5.42)	0.056
IV	18 (60.0)	51 (51.5)		
**Alcohol drinking**				
Non-drinker	3 (10)	32 (32.3)	Referent	
Drinker	27 (90)	67 (67.7)	4.10 (1.16-14.56)	0.029
Light to moderate	2 (6.7)	10 (10.1)	2.13 (0.30-15.12)	
Heavy	25 (83.3)	57 (57.6)	4.68 (1.26-17.44)	0.009
**Betel quid chewing**				
Non-chewer	17 (56.7)	33 (33.3)	Referent	
Chewer	13 (43.3)	66 (66.7)	0.37 (0.16-0.84)	0.018
Light to moderate	4 (13.3)	31 (31.3)	0.25 (0.07-0.87)	
Heavy	9 (30.0)	35 (35.4)	0.50 (0.19-1.30)	0.108
**Cigarettes smoking**				
Non-smoker	3 (10)	18 (18.2)	Referent	
Smoker	27 (90)	81 (81.8)	1.87 (0.51-6.86)	0.348
Light to moderate	21 (70)	60 (60.6)	2.10 (0.55-7.97)	0.528
Heavy	6 (20)	21 (21.2)	1.71 (0.37-8.05)	
**Number of exposure**				
None	1	7	Referent	
1	1	10	0.70 (0.03-14.35)	
2	18	42	3.00 (0.33-27.09)	
3	10	40	1.75 (0.19-16.29)	0.680

*Abbreviation*: *BMI* body mass index, *H&N* head and neck, *NA* not applicable, *OR* odds ratio, *CI* confidence interval.

^*^Including low-grade, high-grade intraepithelial neoplasia and invasive carcinoma; ^+^ Risk assessment every 1-kg/m^2^ increment.

Note that the lifestyle risk factors were recorded according to the frequencies (alcohol on a weekly basis where one time indicates at least 15.75 gm of ethanol: 0, never; 1, once; 2, once to twice; 3, 3–4 times; and 4, ≥5 times; betel quid on a piece per day basis: 0, never; 1, <1 piece; 2, 1–10 pieces; 3, 11–20 pieces; and 4, >20 pieces; cigarettes on a pack per day basis: 0, never; 1, <0.5 pack; 2, 0.5–1 pack; 3, 1–2 packs; and 4, >2 packs) and duration (0, never; 1, <1 year; 2, 1–10 years; 3, 11–20 years; and 4, >20 years). The cumulative lifetime exposure was calculated by multiplying the frequency and duration, and further categorized into these three levels: level 1, never (0); level 2, light to moderate (1–11); and level 3, heavy (≥12).

### Endoscopic examinations by IEE

**Figure 1:**
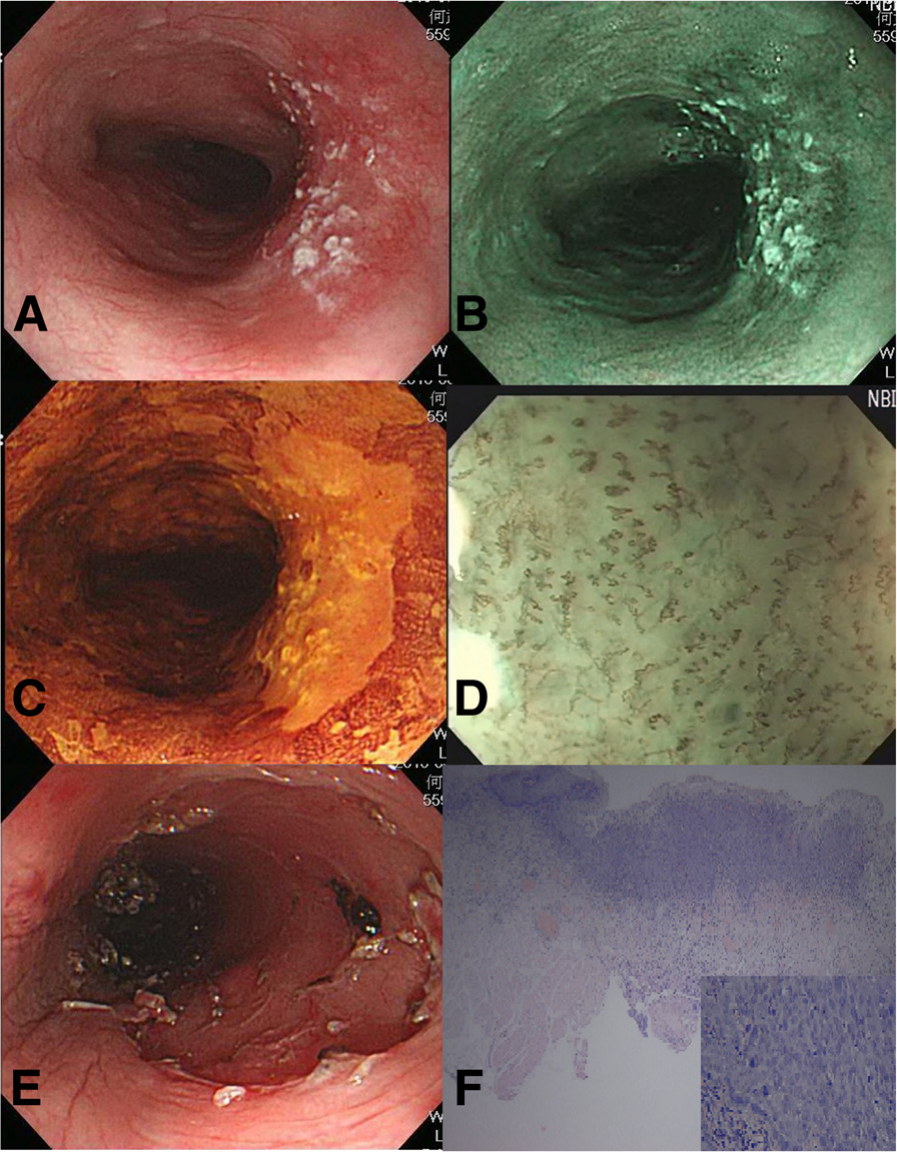
**Endoscopic surveillance and management of synchronous high-grade intraepithelial neoplasia of esophagus in a laryngeal cancer patient. A**, A flat superficial neoplasia with hyperemia in white-light imaging system. **B**, A superficial neoplasia with brownish discoloration under narrow-band imaging system. **C**, Lugol-voiding of the neoplasia after spraying a 1.5% Lugol’s solution. **D**, Abnormal intraepithelial capillary loops under narrow-band imaging system with magnifying endoscopy. **E**, Endoscopic submucosal dissection of the superficial neoplasia. **F**, Mucosal cancer invading the lamina propria (main picture: HE stain, 40x; right bottom: HE stain, 100x).

All of the patients received endoscopic examinations with NBI and ME which has powerful 80 times optical magnification (EVIS LUCERA CLV-260NBI, GIF-H260Z endoscopy, Olympus Medical Systems Corp, Tokyo, Japan), and chromoendoscopy with Lugol’s solution (Sigma-Aldrich, St. Louis, Missouri, USA). All endoscopic examinations were performed by one well-trained endoscopist (C.-S. C.). First, the oral cavity, oropharynx and hypopharynx were evaluated under NBI system [14,15]. The nasopharynx was not examined by endoscopy, but by otolaryngologists. Secondly, the esophagus was examined by white-light imaging (WLI) endoscopy from the esophageal inlet to the esophagogastric junction, and then repeatedly evaluated backward under NBI-ME. After NBI examination, we switched back to WLI and sprayed 10 to 20 mL of 1.5% Lugol solution evenly over the mucosa from esophagogastric junction to upper esophagus. The criteria of suspected lesions were defined as a hyperemic change, uneven or nodularity of mucosa under WLI system (Figure 1A), or brownish discoloration of mucosa with abnormal epithelial capillary pattern (Inoue’s Classification type III ~ V) under NBI-ME system (Figure 1B and 1C) [9,10,16,17], or a well-demarcated Lugol-unstained area (Figure 1D) with a diameter greater than 5 mm or any Lugol-voiding lesions accompanied with pink-silver sign which is often associated with high-grade neoplasia [18]. Finally, the stomach, and the first and second portion of the duodenum, were examined under WLI using the typical panendoscopic procedure. Endoscopic biopsy was done for all suspected lesions fulfilling the criteria mentioned above with histological results served as reference standard.

### Histopathology and decision making of the treatment strategy

The biopsied tissues were examined by an experienced pathologist (Y.-H.C.) and classified by the revised Vienna classification of epithelial neoplasia [19]. Chronic inflammation and squamous hyperplasia belong to the diagnosis of indefinite for neoplasia. Esophageal invasive carcinoma and squamous dysplasia which are associated with increased risk for developing malignancy were included for risk analysis [20]. The 7th edition of the American Joint Committee on Cancer (AJCC) and the International Union for Cancer Control tumor-node-metastasis system was used for tumor staging [21], and the treatment planning for head and neck cancer patients was made by tumor board review. After a complete review of the medical condition of each patient and the information from local findings, endoscopic and radiological examinations, the final treatment option for H&N cancer patients were discussed and made by gastroenterologists, radio-oncologists, surgical and medical oncologists.

### Statistical analysis

Continuous variables were expressed as mean ± standard deviation and the comparisons between groups were performed using the Student *t*-test; categorical variables were summarized as count (%) and the comparisons between groups were made using the χ^2^ or the Fisher’s exact test when appropriate. Univariate and multivariate logistic regression models were performed for evaluation of the demographic and carcinogenic risk factors for synchronous esophageal neoplasia in H&N cancer patients. Cochrane-Armitage trend test was used for the investigation of the dose–response cancer risk predicting power of carcinogens. The sensitivity, specificity and accuracy of different IEE methods to detect esophageal neoplasia were calculated according to the pathological findings served as standard reference. A two-tailed *p* value <0.05 was considered as statistically significant. The statistical analysis was performed using STATA software (version 11.0; Stata Corp, College Station, TX, USA).

## Results

The demographic characteristics of the study population and risk assessment by univariate logistic regression analysis of these factors are shown in Table 1. A total of 122 male and 7 female head and neck cancer patients were enrolled. Sixty defined suspicious esophageal lesions were biopsied from 53 (41.1%) patients after IEE screening and 30 (23.3%) patients had the presence of dysplastic or cancerous lesions by histopathological examination.

Compared with H&N cancer patients without synchronous esophageal neoplasia, the mean age and sex ratio of those with synchronous esophageal neoplasia were not significantly different. However, the BMI is lower in the group with synchronous esophageal neoplasia, and every 1-kg/m^2^ increment is associated with 0.86-fold lower risk (*p* value = 0.008) for esophageal neoplasia (Table 1). The frequency of patients with presence of synchronous esophageal neoplasia was higher in those with hypopharyngeal cancer (12/32, 37.5%) and laryngeal cancer (4/8, 50%) than those with oral cavity cancer (8/63, 12.7%) and oropharyngeal cancer (6/22, 27.3%) (Table 1). The advanced stages, including 7th AJCC TNM stage III and IV, were associated with increased risk (OR 2.30, 95% CI 0.98-5.42) for esophageal neoplasia, but not statistically significant (*p* value = 0.056). Regarding the three common carcinogen exposures, only drinking alcohol was associated with a higher risk (OR 4.10, 95% CI 1.16-14.56, *p* value = 0.029) of esophageal neoplasia with a stepwise dose–response relationship (*p* value for trend = 0.009). Betel quid chewing was found to be associated with a lower risk (OR 0.37, 95% CI 0.16-0.84, *p* value = 0.018) of synchronous esophageal lesions. Compared with the proportion of betel quid chewers in oral cavity cancer subgroup, there were less betel quid chewers in the hypopharyngeal (48.4% *vs.* 68.3%, *p =* 0.03) and laryngeal cancer (25.0% *vs.* 68.3%, *p =* 0.01) subgroups (not shown in Table 1). The number of concomitant carcinogens used was not associated with an increased risk for esophageal neoplasia.

In the multivariate logistic regression model (Table 2), age, gender, cigarette smoking, betel quid chewing and location of index H&N tumor were not associated with the risk for synchronous esophageal neoplasia. However, status regarding the drinking of alcohol (OR 5.90, 95% CI 1.23-26.44, *p* = 0.020), lower BMI (every 1-kg/m^2^ increment with OR 0.87, 95% CI 0.76-0.99, *p* = 0.036), and advanced stages (stage III&IV *v.s.* I&II) of index H&N cancers (OR 2.98, 95% CI 1.11-7.99, *p* = 0.030) were associated with higher risk for esophageal neoplasia.

**Table 2:** Multivariate logistic regression model for risk assessment^*^

**Variables**	**OR (95% CI)**	***P-*** **value**
**Age** (≧55 *vs.* <55)	1.62 (0.59-4.44)	0.350
**Gender** (male *vs.* female)	0.17 (0.01-2.00)	0.161
**BMI** (1-kg/m^2^ increment)	0.87 (0.76-0.99)	0.036
**Stage of H&N cancer** (III&IV *vs.* I&II)	2.98 (1.11-7.99)	0.030
**Alcohol drinking**	5.90 (1.23-26.44)	0.020
**Betel quid chewing**	0.59 (0.21-1.65)	0.318
**Cigarettes smoking**	1.39 (0.29-6.60)	0.679
**Hypopharyngeal cancer**	1.52 (0.54-4.27)	0.423
**Laryngeal cancer**	4.41 (0.80-24.24)	0.088

*Abbreviation*: *H&N* head and neck, *OR* odds ratio, *CI* confidence interval.

^*^Risk assessment for esophageal low-grade, high-grade intraepithelial neoplasia, and invasive carcinoma.

The characteristics of the esophageal lesions detected by IEE screening are summarized in Table 3. 15%, 43.3% and 41.7% of the lesions were found at the upper, middle and lower third of the esophagus, respectively. Among them, 23.3% and 20.0% were high-grade intraepithelial neoplasia (HGIN) and invasive submucosal carcinoma, respectively. Three esophageal lesions were Barrett’s esophagus presented with intestinal metaplasia. To detect low-grade intraepithelial neoplasia (LGIN), HGIN and invasive carcinoma, the diagnostic performance of NBI system with ME examination which had the sensitivity, specificity, and accuracy of 97.3%, 94.1% and 96.3%, respectively, was better than those of WLI system and LC (Table 3). Seven patients (13.2%) had multifocal esophageal lesions.

**Table 3:** Characteristics of esophageal lesions and diagnostic performance of endoscopy

**Esophageal lesions**	**No. (%)**	
**Histopathology**		
Total biopsied lesions	60 (100.0)	
Chronic inflammation	3 (5.0)	
Squamous hyperplasia	17 (28.3)	
LGIN	11 (18.3)	
HGIN	14 (23.3)	
Invasive carcinoma	12 (20.0)	
Others	3 (5.0)	
Detection of neoplasia^*^	**No. (%)**	**Sensitivity/ Specificity/Accuracy (%)**
**Endoscopy/ Histopathology**	60/37 (100)	-
WLI	24/19 (51.4)	51.4 / 78.3 / 61.7
NBI-ME	37/36 (97.3)	97.3 / 94.1 / 96.3
LC	48/36 (97.3)	97.3 / 52.2 / 81.7
**Location**		
Upper third	9 (15.0)	
Middle third	26 (43.3)	
Lower third	25 (41.7)	
**No. of patients with synchronous lesions**		
Single	46 (86.8)	
Multifocal	7 (13.2)	

*Abbreviation*: *WLI* white-light imaging, *NBI-ME* narrow-band imaging system with magnifying endoscopy, *LC* Lugol’s chromoendoscopy, *LGIN* low-grade intraepithelial neoplasia, *HGIN* high-grade intraepithelial neoplasia.

^*^Detection of low-grade, high-grade intraepithelial neoplasia and invasive carcinoma by histological confirmation.

The treatment strategy had been modified in a total of 20 (15.5%) H&N cancer patients after the IEE examination. The number needed to screen for synchronous esophageal neoplasia to have a modified treatment strategy was 6.45(1/15.5%, 95% CI = 4.60-10.90). The characteristics of these patients are shown in Table 4. Among them, 4 patients had oral cavity cancer, 4 oropharyngeal cancer, 9 hypopharyngeal cancer, and 3 laryngeal cancer. For the staging of the head and neck cancer, there were 4, 4, 4 and 8 patients with stage I, II, III and IV, respectively. The esophageal lesions of these patients were four LGINs, four HGINs, and twelve invasive carcinomas (3, 5, 1 and 3 patients with stage IA, IB, IIA, and IIIA, respectively). Adding esophageal neoplasia into consideration for treatment planning, five patients received extended radiation field involving the esophageal lesions, six patients received esophagectomy, and nine patients were suggested for endoscopic treatment of the early esophageal neoplasia, including radiofrequency ablation, endoscopic mucosal resection or submucosal dissection (Figure 1E and 1F).

**Table 4:** Tumor board review of H&N cancer patients with modified treatment strategy after IEE screening

**No.**	**H&N cancer / TNM stage**	**Location / size (cm) / pathology (TNM stage) of esophagus**	**Treatment strategy without IEE screening**	**Treatment strategy with IEE screening**
1	Oropharynx / II	Lower third / 0.3 / LGIN	Tumor excision	Tumor excision + EMR of esophageal lesion
2	Hypopharynx / I	Middle third / 0.5 / LGIN	Tumor excision	Tumor excision + EMR of esophageal lesion
3	Oropharynx / III	Lower third / 0.5 / LGIN	Tumor excision + LN dissection + Adjuvant CCRT	Tumor excision + EMR of esophageal lesion + Adjuvant CCRT
4	Hypopharynx / III	Middle third / 6.0 / SCC (IIA)	Tumor excision + LN dissection + Adjuvant CCRT	Tumor excision + Adjuvant CCRT with RT field involving esophagus
5	Larynx / I	Middle / 1.5 / HGIN	Laryngectomy	Laryngectomy + ESD of esophageal lesion
6	Larynx / I	Upper / 6.0 / SCC (IB)	Laryngectomy	Laryngectomy + Esophagectomy + Adjuvant CCRT
7	Oropharynx / IVA	Upper / 0.6 / SCC (IA)	Neoadjuvant CCRT + Tumor excision + LN dissection	Neoadjuvant CCRT + Tumor excision + Esophagectomy
8	Oropharynx / II	Middle / 4.0 / SCC (IA)	Tumor excision + Adjuvant CCRT	Tumor excision + Esophagectomy + Adjuvant CCRT
9	Hypopharynx/IVA	Middle / 2.0 / HGIN	Tumor excision + LN dissection + Adjuvant CCRT	Tumor excision + LN dissection + RFA of esophageal lesion + Adjuvant CCRT
10	Hypopharynx / IVB	Upper / 1.5 / SCC(IB)	Definitive CCRT	Definitive CCRT with RT field involving the esophagus
11	Hypopharynx/III	Middle / 5.0 / SCC (IIIA)	Tumor excision + LN dissection + Adjuvant CCRT	Neoadjuvant CCRT + Tumor excision + LN dissection + Esophagectomy
12	Hypopharynx / IVB	Middle / 1.5 / SCC (IIIA)	Definitive CCRT	Definitive CCRT with RT filed involving the esophagus
13	Oral cavity / II	Lower / 0.2 / LGIN	Tumor excision	Tumor excision + EMR of esophageal lesion
14	Hypopharynx / III	Middle / 0.8 / HGIN	Tumor excision + LN dissection + Adjuvant CCRT	Tumor excision + LN dissection + ESD of esophageal lesion + Adjuvant CCRT
15	Oral cavity / I	Upper / 2.0 / SCC ( IB)	Tumor excision	Tumor excision + Esophagectomy
16	Oral cavity / II	Middle / 1.0 / SCC (IA)	Tumor excision	Tumor excision + EMR of esophageal lesion
17	Oral cavity / IVB	Middle / 2.0 / SCC (IIIA)	Definitive CCRT	Definitive CCRT with RT field involving esophagus
18	Hypopharynx / IVA	Upper / 2.0 / SCC (IB)	Tumor excision + LN dissection + Adjuvant CCRT	Tumor excision + LN dissection + Adjuvant CCRT with RT field involving esophagus
19	Hypopharynx / IVA	Middle / 0.8 / HGIN	Tumor excision + LN dissection + Adjuvant CCRT	Tumor excision + LN dissection + EMR + Adjuvant CCRT
20	Larynx / IVA	Middle / 6.0 / SCC (IB)	Laryngectomy + Adjuvant CCRT	Laryngectomy + Esophagectomy + Adjuvant CCRT

*Abbreviation*: *CCRT* concurrent chemoradiotherapy, *EMR* endoscopic mucosal resection, *ESD* endoscopic submucosal dissection, *H&N* head and neck, *HGIN* high-grade intraepithelial neoplasia, *IEE* image-enhanced endoscopy, *LN* lymph node, *LGIN* low-grade intraepithelial neoplasia, *RFA* radiofrequency ablation, *SCC* squamous cell carcinoma.

## Discussion

The development of second primary malignancy of esophagus, H&N region or lung in UADT cancer patients is high [4,5,7,22,23]. A nationwide study in Taiwan, where the prevalence of drinking alcohol, cigarette smoking and betel quid chewing is high, has shown that the standardized incidence ratio (SIR) of esophageal cancer in patients with head and neck cancer is significantly increased [4]. Supported by the concept of “field carcinogenesis” [2], cancer may develop synchronously or metachronously in the UADT. Moreover, the survival rate of those with second primary tumor, especially with esophageal cancer, is significantly lower (5-year survival rate only 6%) than those without second primary malignancy [7,24]. Therefore, it is important to perform surveillance of the esophagus in H&N cancer patients to improve overall outcome. In this study, we have found that drinking alcohol, lower BMI, and advanced stages of the index H&N cancer were associated with a higher risk for synchronous esophageal neoplasia, and the endoscopy examination by the NBI system with ME may be the best screening modality available.

Carcinogen consumption largely contributes to the development of the UADT malignancy, including the cancer of H&N region and the esophagus [1]. Among the common psychoactive substances, alcohol, tobacco and betel nut are well-known carcinogens for the squamous cell carcinoma of H&N region and the esophagus [1,11]. In our study, we have found that only drinking alcohol was the independent risk factor for synchronous esophageal neoplasia by univariate and multivariate analyses (Table 1 and 2). Cigarette smoking and betel quid chewing were not associated with higher risk for synchronous second primary tumor of the esophagus in multivariate logistic regression model. Besides, higher cumulative doses of alcohol lead to a higher risk for esophageal lesions (Table 1). Acetaldehyde, the intermediate metabolite of the ethanol, is mainly responsible for the systemic carcinogenic effect of alcohol [25,26]. The systemic effect of cigarette smoking and betel quit chewing is not as conspicuous as ethanol; by contrast, the inhaled tobacco smoke and topical contact of arecoline in betel nut contributes to the cancer of the respiratory tract, oral cavity and pharynx predominantly, rather than the esophagus [7,25-27]. This hypothesis could explain the reason why drinking alcohol, but not cigarette smoking or betel quid chewing, is associated with a higher risk for esophageal neoplasia in H&N cancer patients. In our study, betel quid chewing was found with lower risk for synchronous esophageal neoplasia in the univariate analysis (Table 1), but without statistical significance by multivariate logistic regression model (Table 2). Because in the univariate analysis, the hypopharyngeal and laryngeal cancers were associated with higher risk of esophageal neoplasia than oral cavity cancer, the interesting finding may result from lower proportion of betel quid chewers in the hypopharyngeal and laryngeal cancer patients than those in oral cavity cancer patients.

Regarding the location of H&N cancers, some researchers have found the tendency of esophageal cancer in patients with oropharyngeal and hypopharyngeal cancers, and the tendency of lung cancer in patients with laryngeal cancer [7,13]. Although the results were based on the hypothesis of the cancerization in respiratory and digestive axes [22], other investigation did not report the similar result [4]. In a nationwide database study [4], the incidence of second primary cancer is higher for esophageal cancer (SIR 8.71, 95% CI 7.55-10.01) than for lung cancer (SIR 1.56, 95% CI1.34-1.80) in laryngeal cancer patients. In our study, the fact that higher proportion of betel quid chewing, which was negatively associated with risk of second primary esophageal neoplasm (OR 0.37, 95% CI 0.16-0.84) for H&N cancer patients, in the oral cancer group than the hypopharyngeal and laryngeal cancer patients might be the reason why the esophageal neoplasia less coexisted with oral cancer patients by univariate analysis, and the locations of index H&N cancer were not an independent risk factor for synchronous esophageal neoplasia in multivariate analysis (Table 1 and 2). Moreover, cancers of hypopharynx and larynx are more closely associated with alcohol consumption than oral cancers [28], and alcohol was highly associated with second primary neoplasia of esophagus in H&N cancer patients by both univariate and multivariate analyses in our result. The underlying pathogenesis relationship between esophageal cancer and different location of H&N cancer is still questionable.

Being overweight is a well-established risk factor for several types of cancers, except for UADT cancer [29]. In a collaborated cohort study conducted in the Asia-Pacific region, the cancer risk and mortality rate were all higher in those with excess bodyweight gain among 424,519 participants, excluding lung and UADT cancer [29]. Another multicenter case–control study conducted in European countries has also revealed the inverse association of BMI gain (BMI changes ≧5%) with UADT cancer risk (OR 0.74, 95% CI 0.62-0.89) [30]. Similar results have been reported in a population-based case–control study [31]. BMI gain of more than 25% was associated with a lower risk for lung cancer (OR 0.53, 95% CI 0.33-0.84) and UADT cancers (OR 0.44, 95% CI 0.27-0.71), especially in cigarette smokers and alcohol drinkers [31]. In our study, we have found that every 1-kg/m^2^ increment in BMI was inversely associated with risk for synchronous esophageal neoplasia (OR 0.87, 95% CI 0.76-0.99) in H&N cancer patients (Table 2). Advanced stage of index H&N cancer was also an independent risk factor for esophageal lesions. Because UADT cancer related symptoms, such as odynophagia, dysphagia or oral pain, may be accompanied with decreased amount of oral intake, a lower BMI is usually observed in UADT cancer patients. However, in our study population, both lower BMI and advanced stage of index H&N cancer were independent risk factors, and most of the esophageal neoplasia detected were early superficial lesions without obstruction signs. This inverse association may indicate the body weight-related tumor biological pathway in cancerization of both H&N and esophageal cancers.

IEE is a useful screening tool for precancerous or cancerous gastrointestinal neoplasia. To detect esophageal neoplasia in high risk population, Lugol’s chromoendoscopy which is one of the dye-based IEE techniques has the sensitivity and specificity about 80.0-96.0% and 63.0-72.2%, respectively [11,32-34]. However, many side effects, such as mucosal irritation, retrosternal chest pain or burning sensation, laryngospasm or bronchospasm, and even cardiac arrest, have been reported [18,35]. These uncomfortable adverse effects and the low specificity hinder the Lugol’s chromoendoscopy from being the screening tool of esophageal neoplasia. On the contrary, NBI system which is one of the equipment-based IEE techniques has a higher sensitivity (88.9%) and specificity (97.2%) for detecting esophageal neoplasia in high risk populations [34]. By pushing a button on the handgrip of the endoscope, esophageal neoplasia with hypervascularity could be easily well-delineated without uncomfortable side effects [9,17]. Moreover, the NBI system is not only applied to the detection of early esophageal neoplasia (accuracy 88.9%), but also superficial cancers of H&N region (accuracy 86.7%) as well [14,15]. With the combination of magnifying endoscopy, the margin and invasiveness of the neoplasia can be predicted accurately [9,16,33,34]. In our study, about 97.3 ~100% of esophageal neoplasia were detected by NBI system with ME and Lugol’s chromoendoscopy. Only 51.4% of the esophageal neoplasia were detected by the traditional WLI system. To screen LGIN, HGIN and invasive carcinoma, NBI systems with ME were shown to demonstrate a better diagnostic performance than both WLI system and Lugol’s chromoendoscopy (Table 3). Thus, routine IEE surveillance of the esophagus, especially using NBI system with ME avoiding the unpleasant side effects from Lugol’s solution, should be part of initial staging of H&N cancer patients.

### Limitations

There were some limitations of this study. First, the sample size of study population was small and it was difficult to perform subgroup analysis for different location of H&N cancer (Table 1). Larger scale investigation is necessary to define H&N cancer patients with highest risk for synchronous esophageal neoplasia. Secondly, the result of this study disclosed the risk factors for synchronous esophageal neoplasia and whether it is true for metachronous lesions was not well-defined. Third, the study was conducted in one single tertiary hospital and all endoscopic examinations were done by one well-trained endoscopist. Although all biopsied suspicious esophageal lesions were defined by single endoscopist, there would be no inter-observer bias in this study. Finally, endoscopic surveillance of esophagus in newly diagnosed H&N cancer patients did change the treatment strategy at a high rate in the result. In the result, four H&N cancer patients (two and one with oropharyngeal cancers at stage II and III, respectively; one with hypopharyngeal cancer at stage I) had synchronous esophageal LGINs (Table 4). Because these esophageal LGINs, which have a relative risk of 2.9 (95% CI 1.6-5.2) for developing malignancy [20], were with small size (≦0.5 cm) and presented in primary H&N cancers at early stage, tumor board decision with curative endoscopic ablation treatment were made. However, whether concomitant management of H&N cancers and synchronous esophageal neoplasia has an impact on the survival still needs longer follow-up study in the future.

## Conclusions

The presence of synchronous esophageal neoplasia in H&N cancer patients is not uncommon. Routine IEE surveillance of the esophagus is very important to initial treatment strategy of newly diagnosed H&N cancer patients, especially for those with alcohol drinking habit, lower BMI, and advanced stage of index H&N tumor. We recommend the routine surveillance of esophagus by performing NBI system with ME examination in the initial staging workup of newly diagnosed N&N cancer patients with risk factors identified by this study, and the treatment of synchronous neoplasia should be taken into consideration.

## Abbreviations

H&N: Head and neck; NBI: Narrow-band imaging; ME: Magnifying endoscopy; OR: Odds ratio; CI: Confidence interval; BMI: Body mass index; UADT: Upper aerodigestive tract; IEE: Image-enhanced endoscopy; WLI: White-light imaging; AJCC: American Joint Committee on Cancer; HGIN: High-grade intraepithelial neoplasia; LGIN: Low-grade intraepithelial neoplasia.

## Competing interests

The authors declare that they have no competing interests.

## Authors’ contributions

1) Conception and design: CSC, LJL, THL. 2) Analysis and interpretation of the data: CSC, LJL, WCL, YHC, YCC, YCL, WFH, PWS, THL. 3) Drafting of the article: CSC, LJL, THL. 4) Critical revision of the article for important intellectual content: CSC, LJL, WCL, THL. 5) Final approval of the article: CSC, LJL, WCL, YHC, YCC, YCL, WFH, PWS, THL. All authors read and approved the final manuscript.

## Pre-publication history

The pre-publication history for this paper can be accessed here:

http://www.biomedcentral.com/1471-230X/13/154/prepub
